# Polygenic risk scores in psychiatry: Will they be useful for clinicians?

**DOI:** 10.12688/f1000research.18491.1

**Published:** 2019-07-31

**Authors:** Janice M. Fullerton, John I. Nurnberger

**Affiliations:** 1 Neuroscience Research Australia, Margarete Ainsworth Building, 139 Barker Street, Randwick, Sydney, NSW, 2031, Australia; 2 School of Medical Sciences, University of New South Wales, High St, Kensington, Sydney, NSW, 2052, Australia; 3 Department of Psychiatry, Indiana University School of Medicine, 355 W. 16th Street, Indianapolis, IN, 46202, USA; 4 Stark Neurosciences Research Institute, Indiana University School of Medicine, 320 W. 15th Street, Indianapolis, IN, 46202-2266, USA

**Keywords:** genetics, psychiatry, polygenic risk scores, clinical practice

## Abstract

Major psychiatric disorders are heritable but they are genetically complex. This means that, with certain exceptions, single gene markers will not be helpful for diagnosis. However, we are learning more about the large number of gene variants that, in combination, are associated with risk for disorders such as schizophrenia, bipolar disorder, and other psychiatric conditions. The presence of those risk variants may now be combined into a polygenic risk score (PRS). Such a score provides a quantitative index of the genomic burden of risk variants in an individual, which relates to the likelihood that a person has a particular disorder. Currently, such scores are quite useful in research, and they are telling us much about the relationships between different disorders and other indices of brain function. In the future, as the datasets supporting the development of such scores become larger and more diverse and as methodological developments improve predictive capacity, we expect that PRS will have substantial clinical utility in the assessment of risk for disease, subtypes of disease, and even treatment response. Here, we provide an overview of PRS in general terms (including a glossary suitable for informed non-geneticists) and discuss the use of PRS in psychiatry, including their limitations and cautions for interpretation, as well as their applications now and in the future.

## Introduction

The concept of *polygenic* risk was initially coined in classic genetics, and it has been discussed and modeled since the early 20th century ^1^. With the advent of genome-wide association studies (GWAS), it has been possible to quantify polygenic risk with some precision in human complex traits, including psychiatric disorders ^2^. *Polygenic risk scores* (PRS) were first applied in psychiatry in 2009 by the International Schizophrenia Consortium ^3^, and the approach has since revolutionized psychiatric genomics research. However, it is not immediately clear—particularly to non-geneticists—how PRS are derived, what their limitations and applicability might be, and how PRS can best be used, now and in the future. This overview is focused toward guiding psychiatric practitioners and trainees, or informed non-geneticists, on the uses, today and in the coming decades, of PRS in psychiatry. Terms in italics are further defined in the accompanying “Glossary”.

## Accelerated knowledge through psychiatric genomics

Early genomic studies in psychiatry assumed that there might be one or a handful of major effect genes or loci (that is, that psychiatric conditions were *monogenic* or *oligogenic*) and that identification of these key risk genes would provide clear insights into disease mechanisms. After decades of studies which failed to find a consistent signal using these simple genetic models, the field moved toward the understanding that most common psychiatric disorders are both *polygenic* and genetically *heterogeneous* (that is, where many tens or hundreds of genetic loci influence disease risk and where the combination of *risk alleles* is different in different people). This was a paradigm shift in the thinking of researchers ^4^; with it came the realization that the sample sizes which were able to be collected by a single investigator or group were not going to provide sufficient power to clearly detect genetic effects, most of which would be quite small individually (though substantial in combination). New and more efficient molecular technologies reduced the costs of genome-wide genotyping, and large-scale highly collaborative approaches—such as those conducted by the Psychiatric Genomics Consortium ^4^ ( http://www.med.unc.edu/pgc)—are now bearing fruit. These large-scale collaborative genomic studies have accelerated our knowledge of the molecular architecture of many psychiatric disorders to an unprecedented degree in the last decade ^5^. These studies have also enabled insights into the *pleiotropy* of genetic effects across disorders ^5^, which are clinically differentiated on the basis of patterns of symptomatology but which often share clinical features.

## An overview of polygenic risk scores

**Figure 1.**
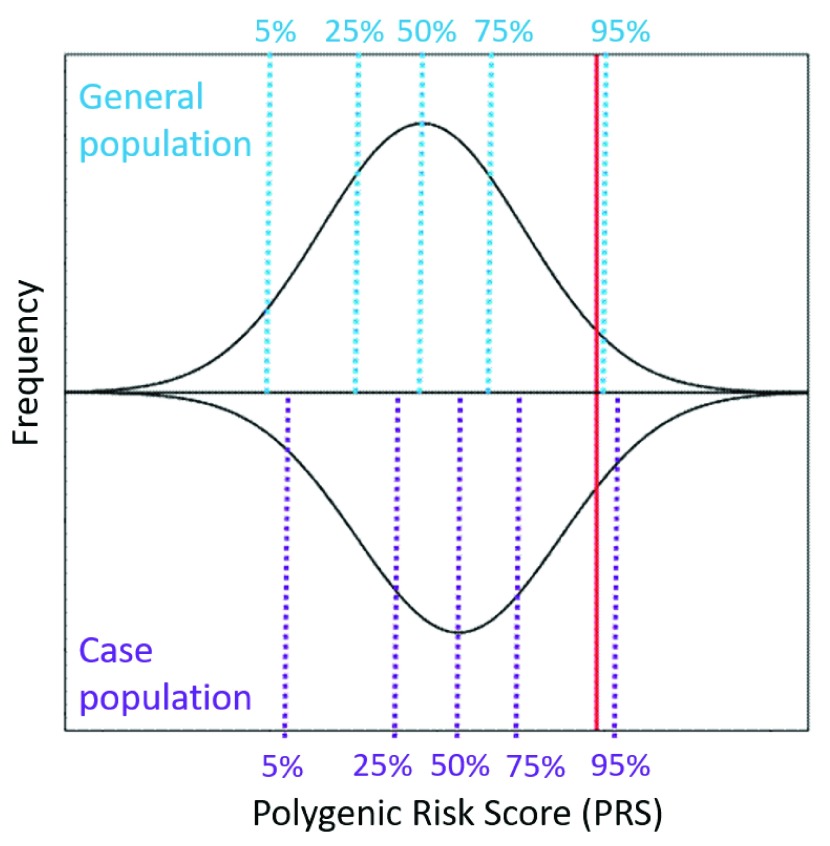
An example of the population distributions of polygenic risk scores (PRS). The black lines represent the distribution of PRS for a complex genetic condition in the (top) general population and (bottom) a case population of individuals affected with the condition. At a population level, the average PRS in the case group is higher than the average PRS in the control group, although many cases will have lower scores than the average person from the general population. The red line indicates a single individual with a “high PRS” – this individual has a score which lies in the top 94% in the general population and the top 90% in case population.

The methods for calculating PRS have been developed in the last 10 years as a tool to capture the cumulative effects of many genetic loci into a single quantitative metric ^2, 3^. This quantitative score sums the effects of individually associated *single-nucleotide polymorphisms* (SNPs) from an independent GWAS, enumerates how many *risk alleles* are carried by that individual at each locus (0, 1, or 2), and weights the risk allele at each locus by its *effect size*. A risk allele is defined as a gene variant that is more commonly found in cases than controls (or that is associated with more severe manifestations of a quantitative trait). Effect sizes are typically estimated as the beta-coefficient for quantitative traits and as an odds ratio for categorical binary traits (with logarithmic transformation of the odds ratios to center values around zero for use in PRS, so that PRS can be computed as a sum of weighted genotypes). An example of a population distribution of a PRS, showing a normal distribution, is illustrated in Figure 1. PRS can be calculated using different sets of disease-associated variants, and typically different *P* value thresholds for disease association are used to create a series of PRSs for a particular disease or trait. The *P* value threshold at which the best distinction is observed between case-control groups (or with variability in a quantitative trait) is selected as optimal. Intuition dictates that only variants that are robustly associated at genome-wide significance contain genuine disease risk predictors and no “noise”; however, such predictors generally explain very little variation in disease risk and therefore have little predictive accuracy ^6^. The common practice, therefore, is to perform genetic prediction by using many *independent risk alleles* across the genome—including many hundreds of thousands of SNP variants, most of which show only weak evidence of disease association on their own—under the assumption that many genuine significant associations are potentially missed because of inadequate power in the original GWAS. This approach yields greater predictive power across many psychiatric disorders, maximizing the predictive power and accuracy for discrimination between cases and controls (as measured by predicted areas under the curve, or AUCs) of around 82% for schizophrenia, 68% for bipolar disorder, 58% for major depressive disorder, and 54% for anxiety ^7^ (compared with an AUC of 50% describing a test with prediction no better than chance), although discrimination estimates vary considerably across cohorts in “leave one out” analyses reflecting independent datasets ^8^. These discrimination estimates will likely improve with more powerful and diverse discovery GWAS ^9, 10^ and improved methods for quantifying polygenic risk. Ideally, in order to be useful, predictive capacity would need to be at least moderately (that is, over 70% to 80%) but preferably highly (that is, over 90%) predictive. Recently, PRS reflecting a selection of SNP sets based on biological processes (for example, signaling pathway membership, protein–protein interactions, or pharmacological treatment response) have been calculated and applied ^11, 12^. These pathway-specific PRS may predict phenotypic variation more robustly than risk scores reflecting overall disorder risk and may be more amenable to mapping of specific *endophenotypes* or phenotypic correlates of disease (such as cognitive and cortical changes which are associated with the disorder or its symptoms) ^13– 15^.

## Limitations and cautions for interpretation of polygenic risk score

In order to effectively use PRS in the practice of psychiatry, it is critically important to understand the limitations of this measure and considerations for interpretation of PRS which we aim to highlight here. First, PRS are highly sensitive to ethnic background—meaning that variability in a PRS can be heavily influenced by allele frequency differences, differences in estimated effect sizes, and differences in population structure across different ethnic groups ^16, 17^. For instance, if a disease-associated risk variant is common in one population but a low frequency in another population (for example, the “A” allele frequency is 20% in the general population of ethnic group X versus 5% in ethnic group Y), then the likelihood of an individual from ethnic group X carrying one or more risk alleles by chance is much higher than for an individual from ethnic group Y (that is, 36% versus 10%, based on the principles of Hardy–Weinberg equilibrium where *f(AA+Aa) = p ^2^+2pq*). Furthermore, owing to differences in population history (including effective population size and distinctive rates of immigration and inbreeding), inheritance patterns of *genomic variants* are more variable and complex in some ethnic groups than others (for example, persons of African ancestry tend to have shorter haplotype blocks compared with persons of European ancestry) ^18– 20^. More complex inheritance patterns will require a greater number of gene variants to be included in the PRS, and because the SNPs included in PRS are often not themselves causal, *causal variants* may be “tagged” by different SNPs in different populations, giving rise to different detected effect sizes and different groups of maximally informative SNPs. Effect sizes are also influenced by gene-by-environment interactions, which may result in population-specific variability. Inclusion of quantitative genetic features of ethnicity as covariates in the association tests will adjust—to some degree—these ethnicity-specific effects in the primary GWAS, but application of those data to independent samples of different ethnicity to the discovery sample is especially prone to errors and fraught with potential misinterpretation ^16, 17^. For this reason (among many others), it is imperative that, rather than applying recent Eurocentric approaches, future GWAS include analyses of subjects from diverse ethnic backgrounds, improving generalizability and utility of these results for all populations ^21^, and efforts are under way to resolve this crucial issue.

Second, the accuracy of the PRS is dependent on the informativity of the discovery sample which is used to define the disease-associated variants and risk alleles. Studies with larger sample sizes yield more power to detect small genetic effects and provide more accurate estimates of the effect sizes of SNPs ^22^. Adequate sample sizes today typically include tens of thousands of cases and a similar or larger number of controls. Sample sizes, and consequently power, are expected to increase over time going forward. However, large sample sizes do inherently carry increased heterogeneity at both clinical and genetic levels and have the potential of making genetic association signals less clear. Samples for large collaborative efforts are often ascertained across multiple investigator sites and in multiple countries, where diagnostic practices and exposure to demographic/epidemiological risk factors might be slightly different, potentially introducing artifactual associations and contributing to fluctuating strengths of association and effect size estimates across cohorts. In general, PRS based on samples which are from the Psychiatric Genomics Consortium and for which summary statistics are publically available have been reliably vetted and processed (but currently are based largely on individuals of Caucasian European descent, the implications of which are described above).

Furthermore, by using information from a GWAS of a second related trait or disease, it is possible to calculate a PRS in a population which has been ascertained with a particular trait or disease in mind. An example would be the use of alcohol consumption GWAS summary statistics (that is, examining units of alcohol consumed per week) in order to characterize risk for alcohol use disorders, which are related constructs but not directly linked. If the genetic causes of those two traits overlap and the predictive performance of the PRS is good, then significant association with the related PRS will also be observed; however, the SNPs providing maximal distinction between case and control groups in the related trait may differ from those which would maximally distinguish the primary trait. Hence, it is important to identify and understand the primary GWAS which was used as the basis to generate the PRS before making a judgment on the accuracy (or lack thereof) of a PRS for a specific analysis or trait. In the example above, alcohol consumption turns out to have only partial genetic overlap with risk for alcohol use disorder and thus the accuracy of prediction is limited ^23^.

Finally, as GWAS typically focus on a single class of genetic variation—the common SNP contributions to disease risk—PRS typically will measure only the contribution of common *single-nucleotide variants* in an individual and not other classes of variation which may also impact genetic risk and be particularly relevant in certain individuals. For instance, an individual may carry one or more *copy number variants* which have a large impact on disease risk which will not be considered in a typical PRS. Also, rare pathogenic alleles are typically excluded from PRS derived from GWAS summary statistics, because GWAS typically include only “common” variants with a population frequency of 1% or more. Furthermore, PRS assumes an *additive effect* of individual risk alleles and does not model complex higher-order interactive (or epistatic) relationships between risk variants, simplifying the genetic model under which PRS are calculated. As our knowledge of the contributions of rare variants increases with large-scale sequencing studies ^24^, we may soon see the development of genomic risk predictions which encompass both common and rare single-nucleotide variants, structural variants, and even *epigenetic* factors and which apply more sophisticated underlying genetic models, including higher-order interactive effects to provide a more complete genomic risk prediction profile representative of the whole spectrum of genomic variation and its biological orchestration.

## What polygenic risk scores can do now

•    Differentiate cases from controls on a population (or group) level ^3, 7, 9, 10^; cases are expected to have a significantly higher mean PRS than controls.

•    Inform research on psychiatric endophenotypes or biomarkers ^13– 15^. We expect that a true biomarker of genetic risk will more likely be present in persons with a high PRS than persons with a low PRS.

•    Provide information about phenotypic correlations ^3, 5, 25, 26^. Phenotypes that are correlated with a diagnosis because of overlapping genetic causes would be expected to be correlated with the PRS for a diagnosis as well. Those that are correlated because of environmental exposures or consequences of treatment would show low PRS correlation.

## What polygenic risk scores cannot do now

•    At present, the PRS is generally not very informative of disease status (that is, case or control) for psychiatric diagnoses at an individual level, although PRS are showing potential for clinical utility ^25, 26^, as has been shown in breast cancer, coronary artery disease, obesity, and diabetes ^27, 28^. Refinement of PRS methods and expansion of the discovery datasets may be expected to change this. Extreme PRS may be informative in some cases now (see below).

•    PRS does not help with genomic testing recommended as standard screening for autism and developmental disorders. The focus in this kind of testing is primarily copy number variants, for which the genomic burden is high in affected individuals ^29, 30^. Rare *pathogenic variants* are also informative and can be identified by whole exome sequencing ^31^. The general recommendation for clinical screening is chromosomal microarray plus Fragile X testing ^32^, although expansion to include PRS from GWAS and integrative genomics approaches may occur in the future ^33, 34^.

•    PRS does not substitute for family history in clinical assessment. Family history is an informative marker of genetic risk and a key piece of information guiding clinical diagnosis and management. However, it is very non-specific, in terms of both individual-level risk prediction as well as disease specificity, particularly in the presence of *heterotypic continuity*, or variable clinical presentations at different ages across the life span ^35^. A predictor based on measured genotypes such as PRS should provide important information additional to family history ^36^, as it has for some non-psychiatric conditions ^37, 38^. However, some families with multiple affected members (and arguably a high genetic load toward illness) may have a low PRS ^6^ – in this instance, disease is likely caused by other variants not measured in the PRS, such as rare pathogenic variants or structural variants, including copy number variants or cytogenetic rearrangements.

## The future of polygenic risk scores

**Figure 2.**
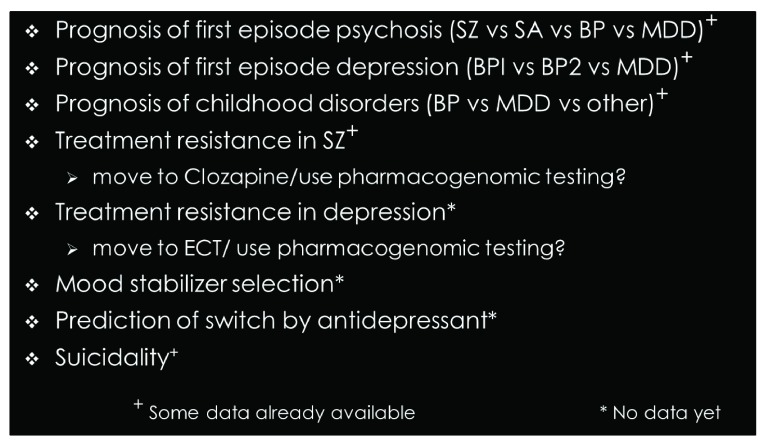
Potential clinical uses of polygenic risk scores. BP, bipolar disorder; BP1, bipolar disorder - type 1, BP2, bipolar disorder - type 2, ECT, electroconvulsive therapy; MDD, major depressive disorder; SA, schizoaffective disorder; SZ, schizophrenia.

Ultimately, the PRS should include actionable information for some individuals, even while many individually associated variants remain to be discovered. This may aid clinicians in the surety of diagnosis, guide implementation of preventative strategies, flag individuals who are at greatest risk of suicide, or enable predictions of long-term prognosis or treatment response ^39– 41^ ( Figure 2). Clinical diagnostics requires high precision (or positive predictive value), where the proportion of individuals who are predicted to be cases (and who truly are cases) is very high. However, the implementation of PRS in clinical practice may also help identify low-risk groups or even treatment-resistant individuals for whom alternative treatment methods—including social and psychological management—may be most beneficial ^42^. Furthermore, PRS may guide patient discussions with genetic counselors to better understand relationships between genetic and environmental risk factors, providing psychotherapeutic benefits and promoting patient empowerment ^43^, and has the potential to reduce stigma and feelings of self-blame in those with established illness, aiding emotional recovery.

A promising method for optimizing PRS was recently reported by Khera *et al*. (2018) ^28^. In this study, three large independent patient samples were used for each of five common medical conditions (coronary artery disease, atrial fibrillation, type 2 diabetes, inflammatory bowel disease, and breast cancer). The first sample was the discovery sample. A GWAS was performed, and a series of PRS was produced by using multiple *P* value thresholds, “pruning” for non-independence, and optimal number of variants for the final dataset. The second sample was used as a validation dataset, in which each of the PRS was applied to find the one that best discriminated cases from controls for that disease. The third sample was the testing dataset, in which only the best validated PRS was used. Using this method, the authors were able to generate good predictive ability (with 80% accuracy for coronary artery disease, for instance). Notably, they were also able to identify subsets of the testing sample with odds ratios of 3, 4, or 5 for having illness. This is comparable to the predictive ability of many monogenic mutations in Mendelian diseases. The group of subjects this applied to was small (for the five diseases, the size of this risk group ranged from the top 1.5% to the top 8.0% of subjects with the highest PRS), but for those groups the PRS might have some clinical utility even now. There is no reason that this method cannot be applied to common psychiatric disorders and it is supported by data showing that subjects with a top-decile PRS for schizophrenia have a threefold increased risk of psychosis ^25^ and a further study showing that the top-decile PRS for major depressive disorder was associated with a 2.5-fold increased risk of major depression ^26^. Of course, the relevant question in a clinical situation is usually not to differentiate between typical cases and symptom-free controls but to identify individual cases who might benefit from specific treatments. We would anticipate that young at-risk individuals with a high PRS might benefit from psychoeducation, introduction of lifestyle changes to reduce stress and promote stability of social and personal rhythms, and increased clinical monitoring and access to support services, particularly as they transition through the peak period of symptom emergence. Early identification of those with an extremely high PRS may eventually lead to early pharmacological treatments. For adult individuals with established illness, specific PRS profiles may potentially indicate those more or less likely to respond to pharmacological treatments or those who would benefit from specialized alternative therapies (such as electroconvulsive therapy).

One might also anticipate that methodological developments in computing PRS may lead to better polygenic risk models and predictors. Competitors to the standard thresholding PRS, incorporating information about underlying linkage disequilibrium structure, are actively being developed (for example, LassoSum, LDpred, and PRS-CS) ^44– 46^. Such improvements may include the incorporation of information about the relationship of genotype to gene expression in the brain, a rapidly developing knowledge base, which would have beneficial effects in optimally weighting and calibrating the calculation of the PRS. In fact, the PsychENCODE Consortium recently showed that incorporation of information from an expression database may increase the predictive power of genotypic data by over threefold ^47^. One may expect a new generation of PRSs based on methods such as this.

Polygenic scores may aid patient stratification and facilitate subphenotyping: for instance, among bipolar disorder cases polygenic scores for schizophrenia have been shown to distinguish schizoaffective cases from others ^48^. A large Psychiatric Genomics Consortium study recently demonstrated that a PRS derived from a study of schizophrenia predicted psychotic symptoms in patients with bipolar disorder ^49^, a finding replicated in an independent study ^50^. A further study showed that schizophrenia PRS was inversely correlated with response to lithium treatment ^41^. Recent research has also shown that PRS may be useful in predicting future psychiatric diagnoses, such that individuals with first-episode psychosis who subsequently developed schizophrenia had significantly different PRS from those who did not become chronically ill, although the discriminative accuracy is not yet sufficient for clinical utility ^51^. Furthermore, higher PRS in patients with first-episode psychosis significantly predicted higher post-treatment symptom scores after 12-week follow-up, indicating that patients with low PRS were almost twice as likely to be treatment responders than patients with high PRS (that is, based on a median split into two categories) ^52^.

Another avenue where PRS may be useful in clinical practice in psychiatry is in the prediction of risk of commonly comorbid general health conditions, which impact treatment options and treatment adherence, as well as long-term health outcomes for people with psychiatric illness. For instance, a depression PRS has been associated with higher risk of ischemic stroke, such that stroke risk increased by 3.0% for every one-standard-deviation increase in PRS for those of European ancestry and by 8% for those of African ancestry ^53^.

## Conclusions

Increasing discovery sample sizes and ethnic diversity for GWAS, potentially leveraging large populations represented in national biobanks and disease consortia, will improve the prediction models for PRS considerably, including improvements in both the positive and negative predictive value ^22, 42^. Other methodological changes in PRS calculation, particularly machine learning approaches which may simultaneously examine both clinical and genetic heterogeneity ^54^, may be expected to have an impact on predictive power as well. Thus, although PRSs are not routinely used in clinical practice in psychiatry at this time, the great advances afforded by current genomics research may reveal avenues for personalized medicine which will drastically change the way that psychiatric disorders are treated clinically in the years to come.

## Glossary

***Monogenic:*** Monogenic disorders are caused by a mutation in a single gene, which has a one-to-one correspondence with disease and is said to be causative. The mutation may be present on one or both chromosomes (where one chromosome is inherited from each parent).

***Oligogenic:*** Oligogenic inheritance represents an intermediary between monogenic inheritance (in which a trait is determined by a single causative gene) and polygenic inheritance (in which a trait is influenced by many genes and often environmental factors as well). It describes a condition where a small number of genes determine the trait, linkage is not detected using Mendelian models, and phenotype–genotype correlation can be improved by inclusion of genotypes from another locus (or loci).

***Polygenic:*** A polygenic disorder is one whose phenotype is influenced by more than one gene. Therefore, polygenic disorders are not inherited as simply as single-gene diseases where there is a one-to-one ratio between inheritance of a disease gene and presentation of the disease. Traits such as height or skin color are examples of polygenic traits and often display a continuous distribution.

***Polygenic risk score:*** A numeric measure of genomic burden of genetic variants which increase risk of disease or variability in a quantitative trait on the basis of variation in multiple genetic loci and their associated effect sizes.

***Heterogeneous:*** Genetic heterogeneity can be defined as mutations or risk variants at two or more genetic loci that produce the same or similar phenotypes. A heterogeneous disorder is a disorder which can be caused by mutations at two or more loci but with the same clinical presentation. In the context of polygenic disease, different combinations of variants in different genes may result in the same or similar clinical presentation.

***Pleiotropy:*** Where one gene (or genetic variant) influences two or more seemingly unrelated phenotypic traits, the gene is described as pleiotropic.

***Genomic variant:*** Genomic variation describes the differences between our genomes, which may or may not have an impact on our health. These can vary in size, and may impact the DNA sequence within protein coding sequence of genes, may lie in introns (i.e. the nucleotide sequence within a gene that is removed by RNA splicing before the mature RNA is formed, and which may be transcribed into protein) or intergenic regions (i.e. the nucleotide sequence between genes). A genome contains millions of genetic variations, or variants, which make each person unique.

***Single-nucleotide polymorphism (SNP) or single-nucleotide variant:*** A very common type of genetic variation, representing a difference in a single-nucleotide building block in the DNA sequence, which may or may not have any effect on gene function or any trait. Each SNP variation is present to some appreciable degree (for example, >1%) within a population.

***Risk allele (or risk variant):*** A risk allele is defined as a gene variant that is more commonly found in cases than controls but is not observed exclusively in either group. This is in contrast to a mutation, which has a one-to-one relationship to disease, and is described as disease-causing.

***Independent risk allele (or risk variant):*** A risk variant which is inherited independently of other risk variants in a population because of random assortment and recombination. Identification of independent risk alleles requires a process known as “pruning”, in which variants close to each other on a chromosome, which are frequently transmitted together and therefore said to be in “linkage disequilibrium”, are removed until the remaining variants are effectively statistically independent.

***Causal variant*:** Causal variants have a direct biological effect and a direct effect on the phenotype. Causal variants are responsible for the association signal at a locus, although the association may be identified by using other non-causal variants in linkage disequilibrium with the causal variant.

***Effect size:*** A quantitative measure of the magnitude of a genetic association, determined by its contribution to the total genetic variance of the trait as well as the statistical power to detect it in an association study. Effect sizes are also influenced by gene-by-environment interactions, which may result in population-specific variability when a specific environmental exposure is more common, increasing the apparent strength of a genetic association. For most types of effect size with a center around zero (for example, a beta-coefficient), a larger absolute value always indicates a stronger effect; the main exception is if the effect size is an odds ratio (which centers around one).

***Additive effect***: Describes the condition where the joint effect of two or more independent variables on an outcome is equal to the sum of their individual effects. This is in contrast to a synergistic (or epistatic) interaction, where the combined effect of two or more independent variables on an outcome is greater than the sum of their individual effects.

***Copy number variant (CNV):*** Copy number variation is a phenomenon in which sections of the genome are deleted, repeated, or inverted and the number of repeats in the genome varies between individuals in the population. CNVs can contain whole genes or crucial regulatory elements which influence gene expression or may have no appreciable functional effect.

***Pathogenic variant:*** A genetic alteration that increases an individual’s susceptibility or predisposition to a trait, disease, or disorder. When such a variant (or mutation) is inherited, the development of symptoms is more likely but not certain.

***Endophenotype:*** Also known as intermediate phenotype, this genetic epidemiology term is used to separate behavioral symptoms into more stable phenotypes, which may reflect more elementary processes with a clear genetic connection. A quantitative biological trait that is reliable in reflecting the function of a discrete biological system and is reasonably heritable may be more closely related to the root cause of the disease than the broad clinical phenotype or complex psychiatric phenomena, typically defined by exceeding a symptom threshold.

***Epigenetic:*** Relating to or arising from non-genetic influences on gene expression (or a heritable phenotype) that do not involve alterations in the DNA nucleotide sequence. Such changes can include chemical modifications of the DNA sequence or changes in chromatin structure which alter the accessibility of the DNA to proteins involved in gene expression, thus providing a mechanism for turning on or off expression in response to environment.

***Heterotypic continuity:*** The prediction of a disorder by another disorder or disease ^55^, where disease A would be the cause of disease B, people with disease B would often present with disease A first, or that disease A and B share a common vulnerability factor.
